# Main roads and land cover shaped the genetic structure of a Mediterranean island wild boar population

**DOI:** 10.1002/ece3.8804

**Published:** 2022-04-06

**Authors:** Roberta Lecis, Olivia Dondina, Valerio Orioli, Daniela Biosa, Antonio Canu, Giulia Fabbri, Laura Iacolina, Antonio Cossu, Luciano Bani, Marco Apollonio, Massimo Scandura

**Affiliations:** ^1^ 9312 Department of Veterinary Medicine University of Sassari Sassari Italy; ^2^ Department of Earth and Environmental Sciences University of Milano Bicocca Milano Italy; ^3^ 1004 Faculty of Mathematics, Natural Sciences and Information Technologies University of Primorska Koper Slovenia; ^4^ 1004 Department of Chemistry and Bioscience Aalborg University Aalborg Denmark

**Keywords:** gene flow, landscape genetics, microsatellites, population structure, Sardinia, *Sus scrofa meridionalis*

## Abstract

Patterns of genetic differentiation within and among animal populations might vary due to the simple effect of distance or landscape features hindering gene flow. An assessment of how landscape connectivity affects gene flow can help guide management, especially in fragmented landscapes. Our objective was to analyze population genetic structure and landscape genetics of the native wild boar (*Sus scrofa meridionalis*) population inhabiting the island of Sardinia (Italy), and test for the existence of Isolation‐by‐Distance (IBD), Isolation‐by‐Barrier (IBB), and Isolation‐by‐Resistance (IBR). A total of 393 Sardinian wild boar samples were analyzed using a set of 16 microsatellite loci. Signals of genetic introgression from introduced non‐native wild boars or from domestic pigs were revealed by a Bayesian cluster analysis including 250 reference individuals belonging to European wild populations and domestic breeds. After removal of introgressed individuals, genetic structure in the population was investigated by different statistical approaches, supporting a partition into five discrete subpopulations, corresponding to five geographic areas on the island: north‐west (NW), central west (CW), south‐west (SW), north‐central east (NCE), and south‐east (SE). To test the IBD, IBB, and IBR hypotheses, we optimized resistance surfaces using genetic algorithms and linear mixed‐effects models with a maximum likelihood population effects parameterization. Landscape genetics analyses revealed that genetic discontinuities between subpopulations can be explained by landscape elements, suggesting that main roads, urban settings, and intensively cultivated areas are hampering gene flow (and thus individual movements) within the Sardinian wild boar population. Our results reveal how human‐transformed landscapes can affect genetic connectivity even in a large‐sized and highly mobile mammal such as the wild boar, and provide crucial information to manage the spread of pathogens, including the African Swine Fever virus, endemic in Sardinia.

## Graphical abstract

Genetic structure and landscape genetics were investigated in the wild boar (*Sus scrofa meridionalis*) population inhabiting the island of Sardinia (Italy). A sharp genetic structure into different subpopulations was observed, after removing the confounding effect of hybridization. Isolation by resistance due to main roads and land cover explained the observed genetic discontinuities.

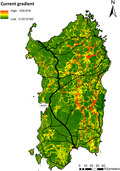

## 1 INTRODUCTION

Land‐use changes can strongly affect the degree of landscape permeability to animal movement and impact genetic differentiation between and within populations of the same species (Lowe & Allendorf, 2010). Moreover, ecological barriers can lead to a disjunction and, sometimes, a complete isolation of subpopulations. The shortage of permeable pathways and the presence of ecological barriers might limit gene flow between subpopulations and contribute to a loss of genetic diversity by genetic drift and to an increase of inbreeding (Balkenhol & Waits, 2009). In the last two decades, several analytical approaches have been developed to infer micro‐evolutionary processes driven by habitat fragmentation and human infrastructures, giving rise to the discipline called landscape genetics (Manel et al., 2003; Storfer et al., 2010). Landscape genetic studies integrate population genetics, spatial analyses, and landscape ecology to test hypotheses about how environmental features influence population genetic structure and gene flow (Storfer et al., 2007). Since urban, suburban development and road network extension are among the primary causes of habitat fragmentation, this analysis can be helpful in planning management practices for species conservation (Holderegger & Di Giulio, 2010; Kimming et al., 2020; Serieys et al., 2014). In fact, several studies indicated that landscape features can shape the gene flow within populations of large mammals (Castilho et al., 2011; Coulon et al., 2006; Pérez‐Espona et al., 2008; Rutten et al., 2019; Sharma et al., 2013, Weckworth et al., 2013), and pointed out that assessing levels of population connectivity is particularly important to inform management practices.

Urbanization and development of large networks of transport infrastructures have rapidly increased in Europe. The impact of anthropogenic barriers and habitat fragmentation on gene flow was investigated in different wild ungulates (Coulon et al., 2006; Dellicour et al., 2019; Frantz et al., 2012; Hepenstrick et al., 2012; Renner et al., 2016; Šprem et al., 2013). However, establishing the real impact of such barriers is challenging, since they could have various levels of permeability depending on the species behavioral characteristics. Frantz et al. (2012) showed how the presence of a motorway could differently affect two ungulate species in Belgium, acting as a barrier for the red deer (*Cervus elaphus*), while apparently not disturbing wild boars (*Sus scrofa*).

The wild boar is an ungulate species native to Europe (Apollonio et al., 2010) and one of the widest‐ranging mammals in the world, adaptable to almost any type of environment. Climate represents the main limiting factor for wild boars, through its effect on physiology and metabolism, or through its indirect effect on food availability and accessibility (Geisser & Reyer, 2005; Melis et al., 2006; Vetter et al., 2015). In the last decades, wild boar populations in Europe have been increasing in numbers and distribution (Massei et al., 2015), causing conflicts with humans, also linked to public health. A major threat arises from the infection of wild boar populations with African swine fever (ASF) virus, which has been endemic in Sardinia since 1978 (Jurado et al., 2018) and spreading within the EU since 2014 (EFSA Panel on Animal Health & Welfare, 2018), with a recent outbreak recorded in north‐western Italy. Spillover of ASF from free‐ranging wild boar to farmed domesticated pigs has been detrimental to the domestic pig industry (Bosch et al., 2020; Reiner et al., 2021). Thus, understanding the spatial behavior of wild boar is essential for managing ASF in the free‐ranging wild boar population.

Wild boars are characterized by a variable use of space (Keuling et al., 2008), regardless of the habitat occupied. Wild boar dispersal takes place between 11 and 16 months of age and usually covers limited distances (<20 km, Keuling et al., 2010; Truvè & Lemel, 2003). Dispersal patterns are influenced by various factors such as population density, habitat structure and quality, and climate (Dardaillon & Bougnon, 1987; Keuling et al., 2010). For instance, wild boar is known to modify its activity and spatial patterns in relation to human disturbance. If undisturbed, wild boars tend to be active during the day, while under hunting pressure and high human disturbance they shift their activity to nocturnal (Brivio et al., 2017; Podgórski et al., 2013). Nevertheless, in some places, wild boars adapt well to human presence and infrastructure in urban areas (Cahill et al., 2012; Osashi et al., 2013).

Our study is focused on the wild boar population inhabiting the Mediterranean island of Sardinia (Italy). Sardinian wild boar, a dwarf form of the European wild boar, is believed to have originated during the Neolithic following a human introduction from the mainland (Albarella et al., 2006). It is currently classified as a distinct subspecies (*Sus scrofa meridionalis* Major 1883), based on both morphological and genetic evidence, as it is characterized by a relevant genetic differentiation, due to its long‐lasting isolation, reported by Scandura et al. (2008), Scandura et al. (2009), Scandura et al. (2011) and Iacolina et al. (2016). However, outdoor pig farming practices in some areas and the uncontrolled release of continental wild boars for hunting purposes have jeopardized the endemic genetic diversity of the population. Scandura et al. (2011), indeed, detected substantial levels of genetic introgression from domestic pigs and continental wild boar, and a relevant population genetic structure into three subpopulations (east, north‐west, south‐west), suggesting that the sharp east‐west genetic differentiation could not be explained by isolation‐by‐distance only, and that landscape features could play an important role.

Here, we analyzed the Sardinian wild boar population, expanding sampling and increasing the number of genetic markers, with the aim to evaluate the genetic structure suggested in the previous studies, in relation to natural and anthropogenic environmental variables that could act as barriers, preventing gene shuffling among subpopulations. Isolation‐by‐Distance (IBD), Isolation‐by‐Barrier (IBB), and Isolation‐by‐Resistance (IBR) were tested using a landscape genetic approach by comparing alternative landscape resistances, as suggested by Balkenhol et al. (2009) and Frantz et al. (2012). Landscape permeability to wild boar movements was tested by combining different genetic clustering methods with landscape resistance modeling.

## 2 MATERIALS AND METHODS

### 2.1 Study area

The island of Sardinia is the second largest in the Mediterranean Sea (24,090 km^2^). Human population density is relatively low for Europe (around 68 inhabitants/km^2^) and people mainly live in major cities and along the coasts, while small villages and large uninhabited areas characterize the interior. Climate is Mediterranean‐temperate at low elevations and along the coast, more continental inland and at higher elevations. Temperature is mild and relatively constant throughout the year (on average 18°C, ranging between a mean of 7°C in winter and 25°C in summer). Annual precipitations range from less than 400 mm in the dry south to 1500 mm in the eastern mountains. Such climatic conditions are suitable for wild boar all over the island.

The island is relatively dry, and some rivers may be reduced to streams in summer. A single small natural lake and several artificial basins are also present, as well as ponds and lagoons along the coasts. Mountains occupy only 13.6% of the territory and are mainly concentrated in the central‐eastern part of the island, reaching a maximum elevation of 1834 m a.s.l. Vegetation is mainly represented by Mediterranean maquis, deciduous forest, grassland, and pastures. Plateaus and flatlands occupy 18.5% of the island territory, the main one being represented by the Campidano plain in the south‐west, a human‐modified landscape dominated by cultivations, especially cereal crops, orchards, and vineyards.

The wild boar is widespread all over the island, occurring in various habitats due to its ecological plasticity, being rare only in the Campidano plain. Estimates of population size are affected by large confidence intervals (a minimum of 20,000 was estimated in 2010), and, based on habitat suitability analyses, higher densities were expected to occur in the central and northern part of the island (Regione Autonoma della Sardegna, 2012).

Human activities and infrastructures potentially have a strong impact on the presence of wild boar. Roads and railway networks, encountered by large mammals, may become an effective barrier, limiting species dispersal, if associated with physical barriers or with high traffic (Kimming et al., 2020). Few main roads with the mentioned features occur in Sardinia: for instance, the SS131 “Carlo Felice”, a motorway with 4 lanes and very few crossing points for wildlife. It crosses the island from south to north along more than 200 km connecting the two major cities, Cagliari and Sassari.

### 2.2 Sample collection and genotyping

**FIGURE 1:**
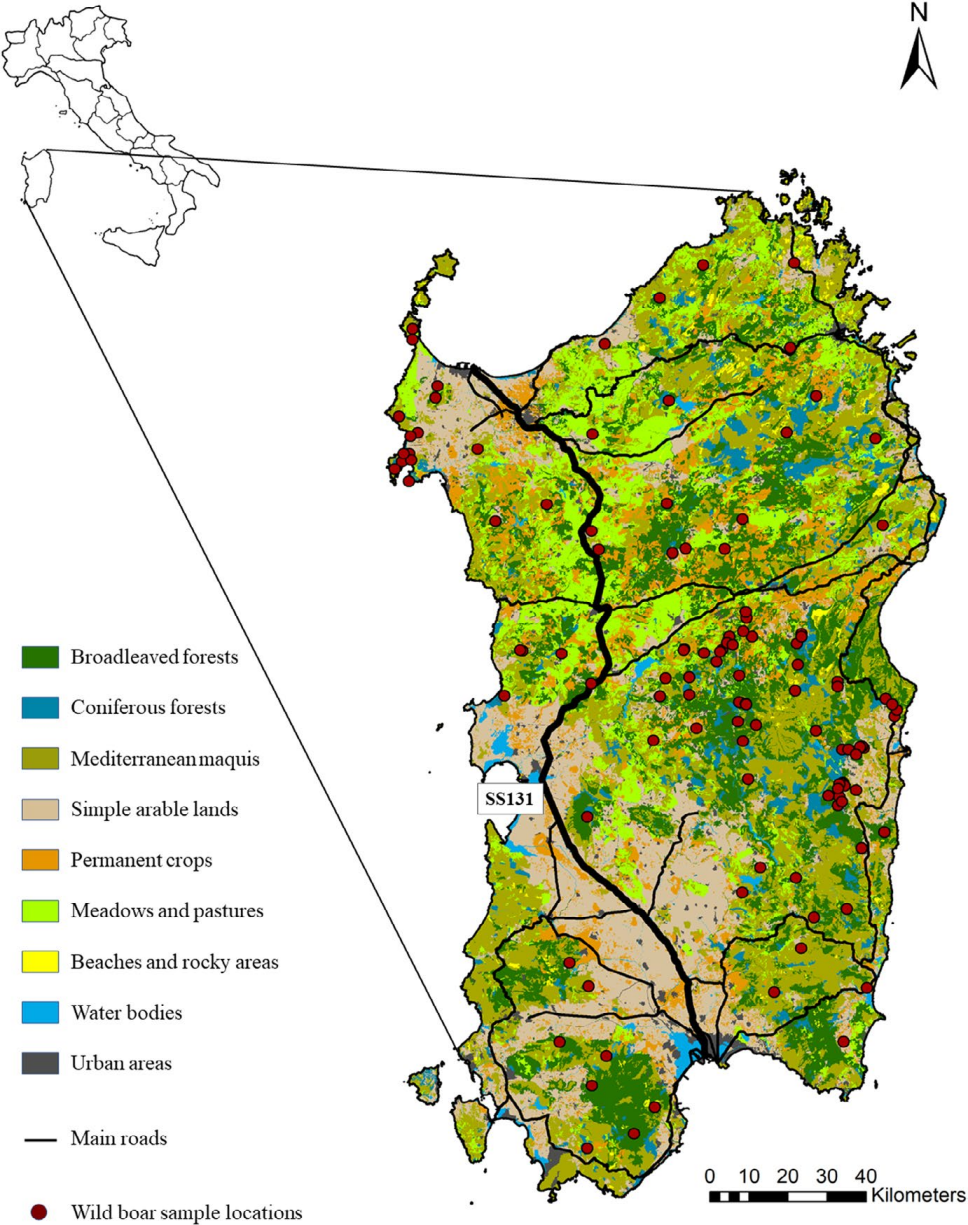
Map of Sardinia showing the geographic locations of the Sardinian wild boar samples and the different land use classes used for modeling. Main roads in the island are shown

A total of 393 wild boar samples were obtained from all over Sardinia by local hunters during the period 2001–2019. Tissue or hair samples were collected from hunted animals and stored, respectively, in absolute ethanol or frozen until analysis. Sampling locations (Figure 1) were mapped using ArcGIS v. 10 (ESRI, Redlands, CA, USA). Accuracy of spatial information differed among samples: in most cases local hunters reported either the municipality or the hunting ground where the animal was found (i.e., polygons in the range 26–547 km^2^, median size 79 km^2^).

DNA was extracted using GenElute kit (Sigma‐Aldrich, St Louis, MO, USA) for tissue samples and Instagene Matrix (Bio‐Rad, Hercules, California, USA) for hair samples, and then stored at −20°C. All samples were genotyped with a panel of 16 microsatellites: S090, SW122, SW2532, S355, SW1492, SW461, IGF1, SW951, SW2021, SW2496, S026, S215, SW72, SW857, S155, and SW24 (details at www.thearkdb.org). Each PCR was performed in a 10 μl reaction volume, containing 3 μl of DNA solution, 0.5 U of Taq DNA polymerase (Euroclone), 1× PCR buffer (Euroclone), 2.5 mM MgCl_2_, 100 μM of each dNTP, and 2 pmol of each primer. Forward primer of each pair was labeled with an ABI fluorescent dye (6‐FAM, HEX, or NED). The amplification profile was set up with an initial step of denaturation at 95°C for 3 min, followed by 35 cycles of 92°C for 45 s, annealing T ranging between 62–52°C for 45 s, and 72°C for 30 s, with a further final extension step of 72°C for 10 min. Amplicons were sized using capillary electrophoresis in an ABI PRISM 3100 or 3730XL Avant automatic sequencer (Applied Biosystems) by the BMR‐Genomics sequencing service (Padova, Italy). Appropriate calibrations were made to standardize microsatellite scoring results when the automatic sequencer was changed. Peak Scanner software v. 1.0 (Applied Biosystems) was used to analyze electrophoretic data.

### 2.3 Microsatellite and population genetic analysis

Data were checked with MICRO‐CHECKER 2.2.3 (Van Oosterhout et al., 2004), in order to detect evidence of null alleles, stuttering or large allele dropout. Deviations from Hardy‐Weinberg equilibrium (HWE) and linkage equilibrium (LE) in the Sardinian wild boar population were tested using GENEPOP v. 4.2 (Raymond & Rousset, 1995). Tests for HWE employed the Markov chain method proposed by Guo and Thompson (2002), with the following chain parameters: 10,000 dememorizations, 100 batches, and 10,000 iterations. Deviations from LE were tested for each pair of loci. Significance levels were lowered, accounting for the number of multiple tests by the sequential Bonferroni procedure (Rice, 1989). Allele frequencies and genetic diversity at the 16 loci, observed (*H_O_*) and expected (*H_E_*) heterozygosity, mean number of alleles per locus (A), and F_IS_ were computed in GENETIX v. 4.05 (Belkhir, 2004).

To ensure that related individuals in the dataset did not bias genetic structure analysis, GENALEX v. 6 (Peakall & Smouse, 2006) and ML RELATE (Kalinowski et al., 2006) were used to estimate pairwise relatedness (QG estimator, Queller & Goodnight, 1989) and to identify the most likely parent‐offspring and full‐sibling pairs in the starting dataset. Only one individual of each pair/group of related individuals was then retained.

The occurrence of imported exotic boars and the signature of genetic introgression from continental populations (Italian peninsula or central Europe) and from domestic pigs in Sardinia was already reported by Scandura et al. (2011). As distortions in allele frequencies due to recent introgressive hybridization can locally alter patterns of genetic structure, we preliminarily screened all Sardinian genotypes to detect and remove individuals showing non‐negligible signals of human‐mediated introgression (see below). For this purpose, Sardinian wild boar genotypes were compared with 100 reference wild boar from different European countries (20 samples from Spain, and 10 samples from France, Austria, Belarus, Croatia, Estonia, Hungary, Luxembourg, and Poland respectively), 50 Italian mainland wild boar, and 100 domestic pigs from Sardinia, including commercial and local free‐ranging individuals. These samples, partially used in previous studies (Canu et al., 2014, 2018; Scandura et al., 2011), had been genotyped using the same protocols as the Sardinian ones.

The full dataset of Sardinian and reference genotypes (*n* = 568) was analyzed by Bayesian cluster analysis in STRUCTURE v. 2.3.4 (Falush et al., 2003, 2007; Hubisz et al., 2009; Pritchard et al., 2000). To detect introgressed individuals, we performed 10 independent Markov chain Monte Carlo (MCMC) runs simulating a number of subpopulations (K) ranging from 1 to 10, with the following settings: admixture model, use population information, correlated allele frequencies, 500,000 burn‐in and 500,000 iterations of data collection. The optimal value of K was determined using the Δ*K* method of Evanno et al. (2005) implemented in Structure Harvester (Earl & VonHoldt, 2012). Accordingly, each individual sampled in Sardinia was assessed in relation to the possible genetic introgression from other wild and domestic populations. Individual admixture was evaluated by referring to the q‐values obtained in the best run with the selected K‐value. To be conservative, only individuals showing >90% cumulative membership to the Sardinian clusters were retained for further analyses (see also Frantz et al., 2013).

STRUCTURE was run again to infer population clustering by analyzing the clean dataset of Sardinian wild boar. A total of 10 independent MCMC runs were performed, simulating a number of subpopulations (*K*) ranging from 1 to 10, with settings: admixture model, no population information, correlated allele frequencies, 500,000 burn‐in and 500,000 iterations of data collection. Again, the optimal *K*‐value was chosen according to the Δ*K* statistics in Structure Harvester (Earl & VonHoldt., 2012). Pophelper (Francis, 2017) was used to edit STRUCTURE results, visualize outputs and produce the final plots.

To confirm the structuring pattern, a Principal Component Analysis (PCA) was also performed using Adegenet package in R v, 4.0.2 (Jombart, 2008; R Core Team, 2020) to detect differentiation among non‐introgressed genotypes in relation to their assigned subpopulation. For this purpose, the purged dataset of “pure” Sardinian wild boar was used, labeling individuals with *q* ≥ 0.6 to a specific Bayesian cluster (from the previous STRUCTURE analysis) as belonging to the corresponding subpopulation. Genotypes were plotted in a two‐dimensional space based on their genetic proximity. Pairwise Rousset's a_r_ genetic distance (Rousset, 2000), shown to be among the most accurate metrics for landscape genetic approaches (Shirk et al., 2017), was computed between Sardinian wild boar samples using SpaGeDi ver. 1.5 (Hardy & Vekemans, 2002).

### 2.4 Landscape genetics analyses

Three potential drivers of genetic variation patterns observed in the Sardinian wild boar population were tested: Isolation‐By‐Distance (IBD), Isolation‐By‐Barrier (IBB), and Isolation‐By‐Resistance (IBR). To assess the relevance of each driver, the resistance optimization process described by Peterman et al. (2014) was implemented using the package ResistanceGA (Peterman, 2018) in R v. 4.0.2 (R Core Team, 2020) within the MARCONI HPC System at CINECA (www.hpc.cineca.it/). This approach uses stochastic search algorithms that solve optimization problems by simulating natural selection processes (Scrucca, 2013) to find the resistance surface values that best explain the observed genetic distances. When applied to categorical surfaces (e.g., land‐cover or barrier maps), the process iteratively creates resistance surfaces assigning new set of resistance values to each category of the map, calculates pairwise ecological (cost) distances from the resistance surfaces, and regresses genetic against ecological distances by fitting linear mixed‐effects models with a maximum likelihood population effects parameterization (MLPE).

The MLPE is used to control for non‐independence among pairwise data (Clarke et al., 2002) and has been recognized as the best performing model in landscape genetic model selection (Shirk et al., 2018). Model performance was assessed through AICc values and optimization proceeded until no additional AICc improvement was obtained. We calculated cost distances among all wild boar sampling locations obtained from the dataset purged from related and/or introgressed individuals (*n* = 270) using Circuitscape 5.0 implemented in Julia (Hall et al., 2021; McRae et al., 2008, 2016). We used the pairwise Rousset's *a_r_* genetic distance as the dependent variable.

To test the IBB hypothesis, we optimized a binary grid surface with a 500 × 500 m resolution where cells crossed by main roads had a value equal to 1 while all other cells had a value equal to 0. Main roads were identified as those with an Average Daily Traffic (ADT) higher than one standard deviation of the mean ADT from all the sampling stations in Sardinia (national traffic monitoring network, http://dati.mit.gov.it/catalog/dataset/traffico‐giornaliero‐medio‐anas). To test the IBR hypothesis, we optimized a categorical land‐cover grid surface. Land‐cover data were obtained from a digital map of Sardinia (Carta della Natura Regione Sardegna, 1:50,000 resolution, Camarda et al., 2015) rasterized at a 500 × 500 m pixel resolution. The original 93 land‐cover classes were reclassified into 9 categories: broadleaved forests, coniferous forests, Mediterranean maquis, simple arable lands, permanent crops, meadows and pastures, beaches and rocky areas, water bodies, and urban areas. Moreover, to have a better representation of the environmental complexity that wild boars face while moving through the landscape, we overlapped the grid surface representing main roads to the land‐cover surface, considering main roads as a further land‐cover type. Thus, IBR simultaneously accounted for the effect of land‐covers and main roads on gene flow. In addition, we assessed Euclidean distance alone (IBD hypothesis) as well as an intercept‐only null model. The relative performances of the IBD model and the optimized IBB and IBR models were evaluated both considering the ΔAICc to the best model and the conditional *R* ^2^ value (*R* ^2^c).

We used the optimized resistance generated by the model with most support to create a current density map of whole Sardinia by following the approach of Koen et al. (2014). We designed a 45‐km‐wide buffer around our study area, roughly 20% of the length and 40% of the width of the study area, then randomly selected 100 nodes around the perimeter of the buffer and used Circuitscape to connect all node pairs. We then removed the buffer and obtained a current density map showing the probability of using each grid cell by free‐ranging wild boars.

In order to integrate information coming from the population structure and landscape resistance analyses, we tested whether the observed genetic clustering of the Sardinian wild boar population into subpopulations can be explained by the landscape resistance among them. Specifically, we regressed the ecological distances calculated using Circuitscape from the optimized resistance surface of the best model on a dichotomous categorical variable that classifies a pair of locations as belonging to the same or to different clusters and the Euclidean distance between them. The latter was included to account for the effect of spatial arrangement of locations in determining genetic clustering and was centered and scaled. Locations that were not assigned to a cluster were removed from the regression model.

## 3 RESULTS

### 3.1 Microsatellite diversity

The total number of alleles detected in the Sardinian wild boar sample was 154, ranging from 6 to 16 per locus and an average of 9.63 ± 3.18 (standard deviation, SD) per locus. Missing alleles represented 2.17% of the dataset. MICRO‐CHECKER did not find any scoring error in the dataset or evidence of allele dropout. Properties of the 16 microsatellite loci used in this study and the variability observed at each locus are shown in Table 1. Referring to the overall sample of Sardinian wild boar (*n* = 393), at all loci observed heterozygosity (*Ho*) was lower than expected heterozygosity (*H_e_*), thus revealing an excess of homozygotes that was confirmed by F_IS_ values. Not surprisingly, GENEPOP analysis detected a significant deviation from Hardy‐Weinberg equilibrium due to heterozygote deficiency in the overall population (all loci *p* < .01), except for locus S026 (*p* = .031) when performing HW test for each locus. Several pairs of loci resulted in linkage disequilibrium (45/120 at α = 0.01, significance corrected for multiple tests).

**TABLE 1:** Genetic diversity at 16 microsatellites analyzed in the Sardinian wild boar (*Sus scrofa meridionalis*) population, sampled between 2001 and 2019 (total sample, *N* = 393)

Locus	Allele size	*A*	*He*	*Ho*	*F*_IS_
S026	82–106	8	0.278	0.260	0.066
S090	228–250	9	0.708	0.604	0.147
S155	145–160	6	0.534	0.438	0.180
S215	137–172	7	0.205	0.124	0.395
S355	242–270	9	0.576	0.361	0.373
IGF1	189–207	10	0.612	0.486	0.207
SW122	111–125	8	0.705	0.565	0.199
SW2532	174–198	11	0.817	0.674	0.175
SW1492	110–128	10	0.758	0.655	0.136
SW461	130–158	13	0.838	0.701	0.163
SW951	111–133	6	0.199	0.122	0.389
SW2021	102–132	14	0.708	0.628	0.114
SW2496	180–228	16	0.833	0.637	0.235
SW72	95–109	7	0.604	0.520	0.139
SW24	79–117	14	0.752	0.605	0.196
SW857	139–155	6	0.572	0.399	0.303
ALL LOCI		*9.625*	*0.606*	*0.486*	*0.198*

Note

*A*, number of different alleles per locus; *H_e_*, expected heterozygosity; *H_o_*, observed heterozygosity; *F*_IS_, inbreeding coefficient.

From the original dataset of 393 Sardinian wild boar samples analyzed with 16 microsatellite loci, 75 individuals showed a high relatedness (i.e., >0.6) to other individuals in the dataset and were likely to represent full‐siblings or parent/offspring. Therefore, they were removed from the dataset to obtain a cleaned pool of 318 unrelated individuals to perform the following analyses.

### 3.2 Identification of introgressed individuals

**FIGURE 2:**
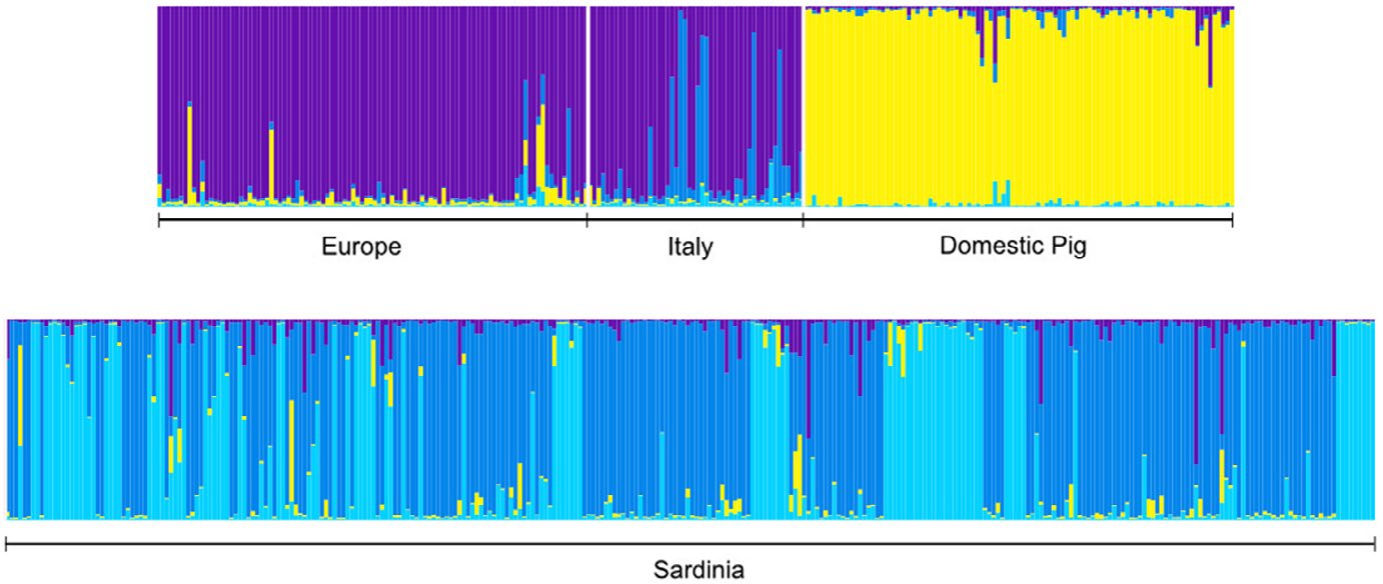
Bar plots illustrating the genetic composition and cluster assignment obtained by STRUCTURE after analyzing 568 samples, including 318 Sardinian wild boars, 100 reference wild boars from different European countries, 50 Italian wild boars, and 100 domestic pigs. *K* = 4 was selected as the best clustering option (see Appendix S1). These results refer to the run showing the highest likelihood, out of 10 replicated runs. Individuals are represented by thin vertical lines, showing the membership (*q*) to the clusters inferred by the program (colored bars). Membership to clusters II and IV (in blue and light blue), both exclusive to the Sardinian population, were pooled. Only individuals univocally assigned to the Sardinian component (q_II+IV_ ≥ 0.9) were identified as non‐introgressed members of the Sardinian population and used for the inference of population structure (*n* = 270)

At *K* = 5 (or higher) the Bayesian analysis in STRUCTURE sharply distinguished the main source populations in the overall sample of 568 individuals (250 reference individuals from mainland Italy, rest of Europe and domestic pigs, and 318 Sardinian wild boar). However, in order to identify individuals with a clear signature of genetic introgression in Sardinia, we selected *K* = 4 as it showed a higher support than *K* = 5 (Δ*K* method, see Figure 2 and Appendix S1), with cluster I identifying European and Italian wild boar, cluster III associated with domestic pigs, and Sardinian wild boar mainly assigned to two clusters (II and IV). Hence, to conservatively assess which individual was a possible recent immigrant/hybrid, we applied the threshold of 0.9 to the sum of q‐values referred to the two Sardinian clusters (qII + IV). For further analyses we thus removed from the dataset a total of 48 (15%) individuals showing introgression from continental wild boar or domestic pigs, and obtained a final purged dataset of 270 Sardinian wild boars.

### 3.3 Genetic structure

**FIGURE 3:**
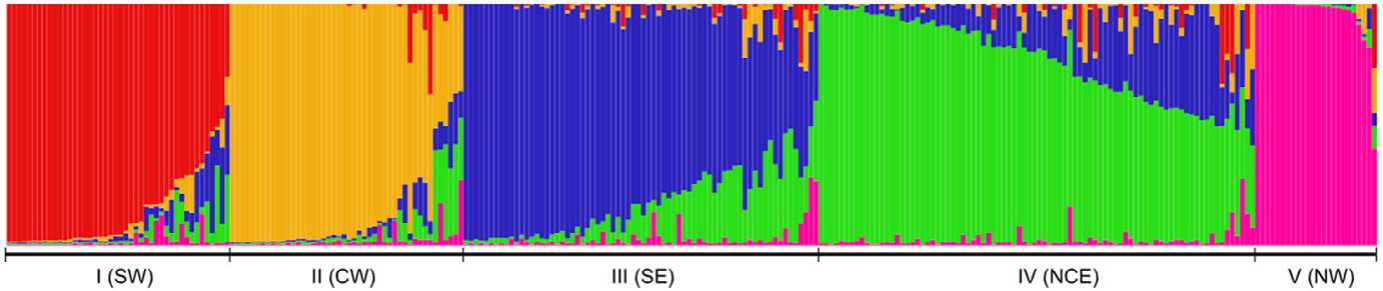
Bar plot illustrating the genetic structure of the Sardinian wild boar population (*n* = 270) inferred by STRUCTURE at *K* = 5. Clusters roughly correspond to five subpopulations: south‐west (SW), central west (CW), north‐west (NW), north‐central east (NCE), and south‐east (SE)

The Bayesian analysis performed in STRUCTURE to highlight the genetic structure of the Sardinian wild boar population (purged dataset) detected a partition in two clusters (*K* = 2), as the most likely, but local maxima were detected also at *K* = 5 and *K* = 8 (Δ*K* method, Appendix S1). At *K* = 2, data suggested a partition between wild boar samples from the west (central and south‐west) and wild boars from the rest of the island (north and east), with the main discontinuity between the two clusters apparently represented by the SS131. At *K* = 5, five subpopulations were clearly identified (Figure 3), corresponding to five distinct geographic areas on the island (see Appendix S2): north‐west (NW), central west (CW), south‐west (SW), north‐central east (NCE), and south‐east (SE).

On the basis of their *q*‐values, 210 out of 270 wild boars (78%) were assigned to one of the five subpopulations with *q* > 0.70, and specifically 23 individuals were assigned to NW, 37 to CW, 39 to SW, 57 to NCE, and 54 to SE. No individual was assigned to a population different from that expected on the basis of its sampling site. Such pattern of population differentiation seemed to identify the presence of genetic discontinuity in coincidence with the SS131 and the Campidano plain. The consistency of results obtained by different analytical approaches points to a sharp structuring in the island with a limited ongoing gene flow between subpopulations.

**FIGURE 4:**
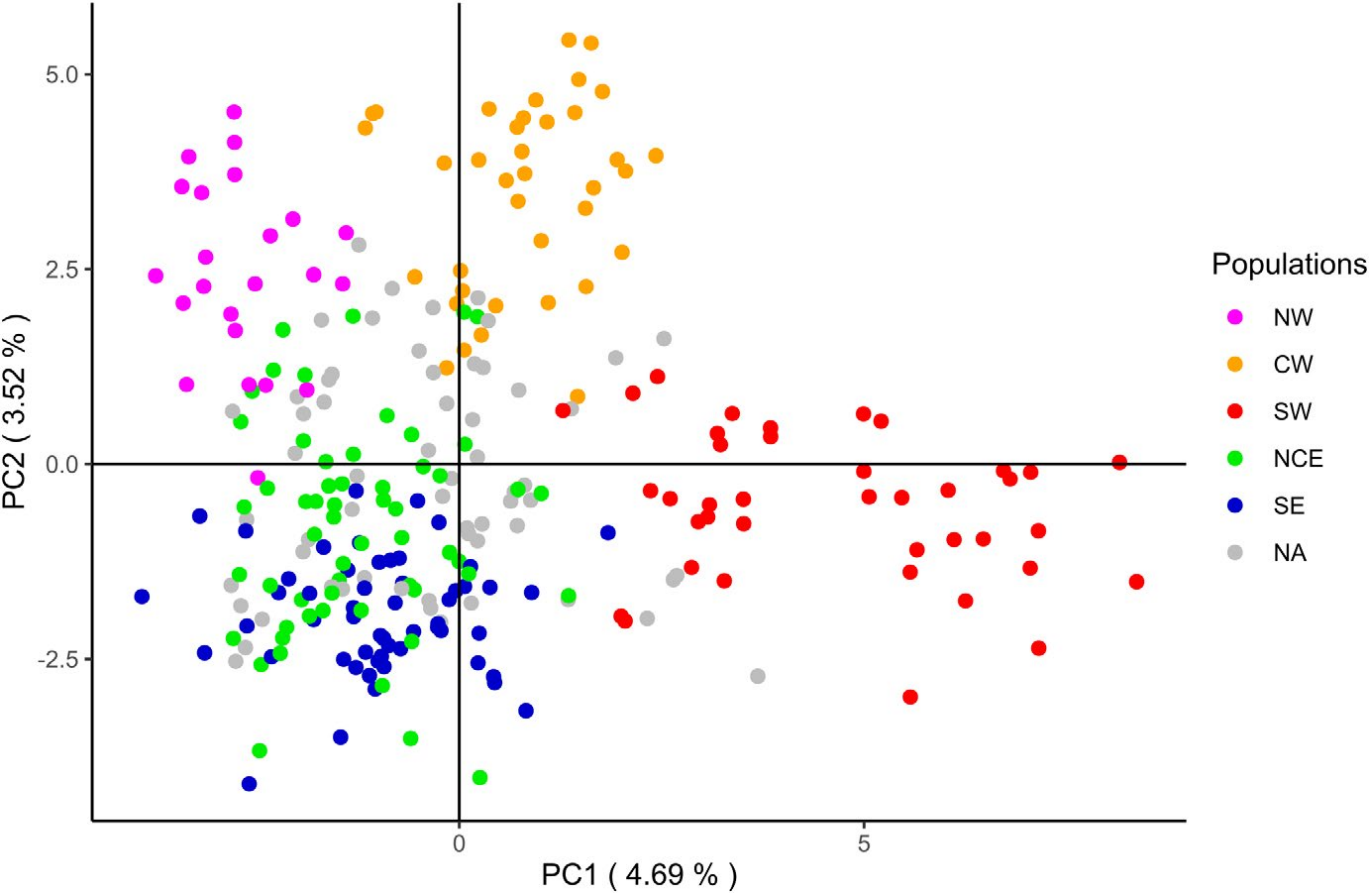
Principal Component Analysis (PCA) plot of 270 Sardinian wild boar performed using Adegenet package in R. The plot show differences among non‐introgressed genotypes in relation to their STRUCTURE‐assigned subpopulation (color): north‐west (NW), central‐west (CW), south‐west (SW), north‐central east (NCE), south‐east (SE), not assigned (NA)

The PCA plot (Figure 4) confirmed the distinctiveness of the three populations geographically identified on the west of the island (NW, CW, SW), while NCE and SE subpopulations overlapped. Some individuals were not assigned to any subpopulation (indicated as grey dots in the PCA, Figure 4).

### 3.4 Landscape genetics

Comparing the best models obtained through the optimization process revealed that the IBR, accounting for land cover and the presence of main roads, was by far the best‐supported hypothesis (Table 2). The IBB had an intermediate performance, but much lower than IBR (ΔAICc = 1510). The IBD hypotheses had a low performance, while the intercept‐only null model had the worst (Table 2).

**TABLE 2:** Comparison of the best models obtained by the optimization processes ran under the Isolation‐By‐Distance (IBD), Isolation‐By‐Barrier (IBB), and Isolation‐By‐Resistance (IBR) hypotheses

Hypothesis	LL	k	AICc	ΔAICc	R^2^c
IBR	25,341	11	−50658	‐	0.613
IBB	24,577	3	−49148	1510	0.461
IBD	24,486	2	−48969	1689	0.460
Null model	21,853	1	−43704	6954	0.262

Note

IBD considers Euclidean distances only; IBB takes into account the presence of main roads as possible barriers; IBR combines the resistance opposed by land cover and main roads.

Abbreviations: AICc, the AICc score; *k*, number of parameters in the model; LL, log likelihood; *R* ^2^c conditional *r* squared; ΔAICc, the absolute value of the difference between the AICc of each model compared to the best performing model.

**FIGURE 5:**
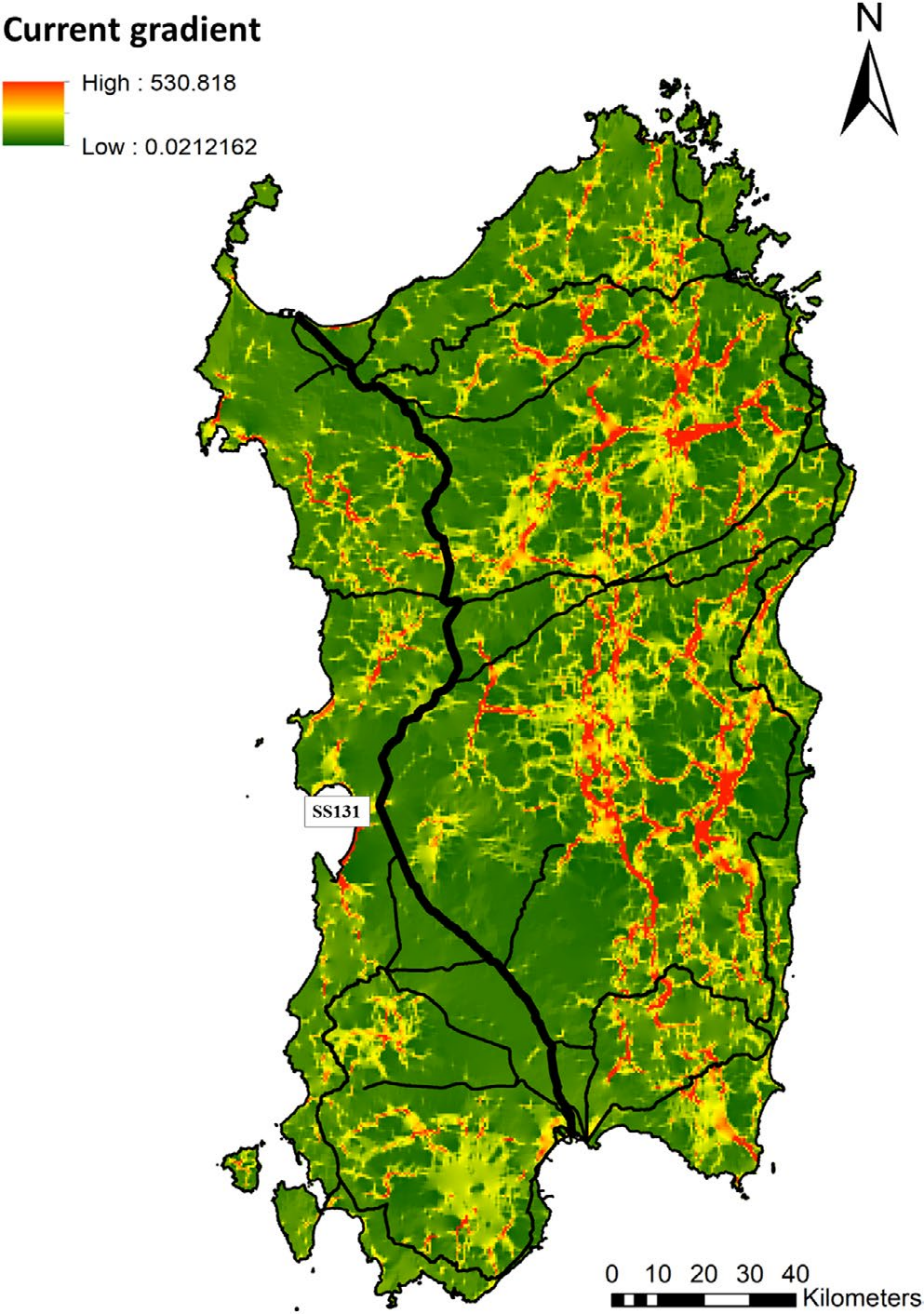
Cumulative current map defined by circuit theory for the best‐supported model (IBR). Resistance values are those shown in Table 3. Main roads appear as solid black lines

The resistance surface associated with the best‐supported model under the IBR hypothesis revealed minimum resistance to wild boar movement in coniferous and broadleaved forests, and water bodies (resistance values <30; Table 3). Meadows and pastures, Mediterranean maquis, and permanent crops showed intermediate resistance values (in the range 1000–2000), while high resistance to movement was assigned to beaches, rocky areas, and simple arable lands (resistance values between 2000 and 3000). However, the highest resistance to wild boar movement was found for urban areas and main roads (resistance values >3000; Table 3). The cumulative current map generated from the resistance surface optimized under the IBR hypothesis is shown in Figure 5.

**TABLE 3:** Resistance values assigned to the different land cover categories (including main roads) by the best‐supported model under the isolation by resistance (IBR) hypothesis

Land cover category	Estimated resistance value
Main roads	3282
Urban areas	3066
Simple arable land	2859
Beaches and rocky areas	2778
Permanent crops	1969
Mediterranean maquis	1167
Meadows and pastures	1035
Broadleaved forests	23
Water bodies	2
Coniferous forests	1

Note

Optimized values refer to the reference category “Coniferous forest”, arbitrarily set at 1.

The regression model testing the effect of Euclidean distance and cluster membership on ecological distances performed well (adjusted *R* ^2^ = 0.624) and confirmed expectations (Table 4). Pairwise ecological distances positively covary with pairwise Euclidean distances (*p* ≤ .001) and were significantly lower in pairs including locations classified in the same cluster than in pairs including locations classified in different clusters (*p* ≤ .001, Appendix S2).

**TABLE 4:** Regression model estimates of the effect of scaled pairwise Euclidean distances (Euc‐dist) and membership to the same genetic cluster (Cluster: same) on pairwise ecological distances

Variable	*β*	SE	*t*	*p*‐value
Intercept	1800.00	4.649	387.21	≤.001
Euc‐dist	715.95	4.558	157.08	≤.001
Cluster: same	−184.50	10.993	−16.78	≤.001

Note

*β* indicates the regression coefficient of Euc‐dist, the difference between mean ecological distance of pairs of locations belonging to the same cluster and those belonging to different clusters (intercept). *β*s were tested against the null hypothesis of being equal to zero. Model adjusted *R* ^2^ = 0.624.

Abbreviations: SE, Standard error; *t*, *t* statistic.

## 4 DISCUSSION

We explored the genetic structure of the Sardinian wild boar population, evaluating the role that landscape features might have played in determining the observed genetic discontinuities. We extended the dataset used in Scandura et al. (2011) by including a larger sample of Sardinian individuals (from 210 to 393) and increasing the number of microsatellite markers (from 10 to 16) and of sampling locations. Our results suggested the presence of five wild boar subpopulations over the island (SW, CW, NW, SE, and NCE), instead of the three subpopulations (ES, NWS, and SWS) previously detected. However, while the partition into three discrete subpopulations on the western side of the island was evident, the subdivision on the eastern side was less obvious. Different statistical approaches, as suggested by Balkenhol et al. (2009) and Frantz et al. (2012), specifically STRUCTURE and PCA, gave support to such genetic structure.

A signature of recent genetic introgression from continental wild boars and domestic pigs was also confirmed (see Scandura et al., 2011). Specifically, gene introgression seemed to mainly affect the eastern and northern subpopulations, while the western ones were marginally affected (Appendix S1). The latter are indeed less affected by the presence of free‐ranging domestic pigs, traditionally raised in open air conditions, and by past releases of captive‐bred or imported boars for hunting purposes. Approximately 15% of sampled individuals were recognized as putative hybrids and their exclusion from population structure analyses prevented the confounding effect possibly arising from the local occurrence of exogenous alleles. After removal of introgressed individuals, as mentioned above, population genetic structure analysis supported a partition into discrete subpopulations. The five clusters identified by STRUCTURE analysis greatly coincided with geographic areas over the island: the north‐western subpopulation (NW) included a small area named Nurra, west to the urban area of Sassari and north of Alghero; the central‐western one (CW) included the areas of Montiferru and Planargia, west of the motorway SS131; the south‐western one (SW) included the areas of Sulcis and Iglesiente, west to the SS131 and to Cagliari urban area. On the other side of the SS131, the two eastern subpopulations, one in the north (Gallura) and center (Barbagia) (NCE), and one in the south, including the area of Sarrabus (SE), showed a weaker genetic divergence between each other. These two areas were included in a single subpopulation by Scandura et al. (2011) and showed a high level of overlap in this study (see Figure 4 and Appendix S2).

IBD, IBB, and IBR were tested to identify environmental and anthropogenic features that might limit gene flow in the Sardinian wild boar population. These analyses revealed that the best‐supported hypothesis was the IBR, assigning a relevant ecological role in hindering Sardinian wild boar movements to main roads, urban areas, and intensively cultivated areas. Euclidean distance alone appeared to barely explain genetic distance, thus confirming results of previous studies at a continental (Scandura et al., 2008; Vilaça et al., 2014) or sub‐continental scale (Niedziałkowska et al., 2021), while contrasting evidence deriving from a few investigations at a regional scale (Frantz et al., 2009, and Goedbloed et al., 2013, in Central‐Western Europe, Alexandri et al., 2017 in Greece).

As discussed by Renner et al. (2016), uncertainty remains as to which landscape features might prevent effective dispersal in wild boars, as different studies showed quite dissimilar results. For instance, Frantz et al. (2012) did not find a major motorway to be inhibiting gene flow in wild boar, while other studies in Germany (Reiner et al., 2021), and the results presented here for Sardinia, suggested that main roads might play a role in creating genetic discontinuities in wild boar populations. Our results also suggested a very different permeability of land‐covers to wild boar movements. The species seems to easily move across broadleaved and coniferous forests as well as through water bodies, which are mainly represented by small lakes and rivers, the latter often reduced or even dried out in summer. Meadows and pastures, Mediterranean maquis, and permanent crops (olive groves and vineyards) showed intermediate permeability to wild boar movements. Mediterranean maquis showed a lower permeability to the species than expected. The dense vegetation structure typical of Mediterranean maquis probably makes this habitat difficult to cross by wild boars and therefore not preferentially used during long‐range movements. Conversely, permanent crops showed a higher permeability than expected offering a relatively suitable environment for species movements, probably due to the presence of both shelter and concentrated, predictable, easily accessible food sources (Torretta et al., 2021).

Finally, main roads, urban areas, and intensive cultivated fields seem to completely prevent gene flow, contributing most to the genetic differentiation within the island population. The role of these landscape features in shaping the genetic clustering observed in the Sardinian population was supported by regression analysis. Genetic similarity between two individuals in the population, and their cluster membership, are well explained by their ecological distance, i.e., the cumulative resistance opposed by landscape elements occurring between the two individuals. The presence of unsuitable habitats and man‐made infrastructures can thus effectively limit the movements of a highly mobile species such as the wild boar. This result suggests that the Sardinian wild boar might not be so generalist regarding habitat preferences for its moving patterns (Dondina et al., 2019). Particularly, the genetic differentiation between western and eastern wild boar subpopulations seemed to occur in conjunction with the motorway SS131 (Figure 1). On the other hand, reduced gene flow between south‐west and eastern areas might be due to the presence of the Campidano plain. In this area, the motorway connects two major urban centers (Cagliari and Oristano), crossing a lowland characterized by suburban and industrial areas scattered in an intensive agricultural territory, thus being fairly unsuitable for the species movements. A similar situation can be found along the Tirso Valley, an agricultural area enhancing the discontinuity between CW and SW populations. Other main roads might partially explain the three clusters identified on the west side, reciprocally isolated (i.e., no recent gene flow). Such barriers to wild boar movements in Sardinia might also have prevented the spread of introgressed genes from the eastern subpopulations to the rest of the island, and probably partially safeguarded the genetic integrity of the western subpopulations (as remarked in Scandura et al., 2011). This would be very interesting from a management and conservation viewpoint, as only negative effects are typically associated with anthropogenic barriers. Although a long time lag is usually expected between a causal factor and a detectable genetic effect, simulations proved that a limited number of generations (as small as 15) can be enough for the genetic signature of a landscape barrier to become detectable (Landguth et al., 2010). Accordingly, studies exploring genetic discontinuities linked to linear barriers have documented the relevant effect of infrastructures built just four decades before (Epps et al., 2005; Hepenstrick et al., 2012; Pérez‐Espona et al., 2008). This time span is similar to that elapsed from the enlargement of Sardinian main roads and the SS131, thus supporting their likely relevant role. Specifically, the SS131 motorway is almost devoid of corridors allowing wildlife crossing for the entire stretch of about 200 kilometers. Up to now, scarce information is available about *Sus scrofa meridionalis* spatial behavior and habitat preferences in Sardinia, therefore we cannot exclude differences with the continental counterparts, which might justify divergent results from those observed in previous studies (i.e., Frantz et al., 2012).

As discussed by Reiner et al. (2021), detecting genetic boundaries associable to landscape elements might also help to improve understanding of population connectivity in order to control the potential introduction and spread of diseases transmitted by wild boars. This would be of growing relevance for pathogens such as African swine fever virus, which is transmissible between domestic pigs, wild boar and hybrids, and represents a big threat to the pig economy worldwide (Busch et al., 2021). ASF has been endemic in Sardinia for many years (Jurado et al., 2018), and new outbreaks have been recently recorded in north‐western Italy (https://www.reuters.com/markets/commodities/african‐swine‐fever‐found‐wild‐boar‐italy‐regional‐government‐says‐2022‐01‐07/).

According to our data, the Sardinian wild boar population should not be managed as a single panmictic unit, rather subpopulations should be treated as separate management units. The lack of gene flow across barriers (e.g., the SS131 and Campidano plain) should be taken into account in the definition of spatial units for disease prevention. Results may also have implications for the management of other wild species in Sardinia. Given that urban areas, main roads and the most intensively cultivated areas apparently play a role as barriers to gene flow in the wild boar population, they could also represent a cause of fragmentation for other mammals (including endemic and endangered species), promoting isolation and genetic drift. However, the effect on other species should be tested by targeted studies, as landscape features might have various impacts on different species (Renner et al., 2016). Concluding, this study confirms how the joint effect of landscape features can generate genetic discontinuities even across a large mammal population, as already observed in other species such as red deer (*Cervus elaphus*, Frantz et al., 2012) and bobcats (*Lynx rufus*, Serieys et al., 2014). Further research would improve knowledge on the role of specific habitat features in preventing an effective dispersal in Sardinian wild boar, although general conclusions about landscape permeability in this species should not be drawn from individual studies (Renner et al., 2016). Finally, possible long‐term detrimental effects (small population size, inbreeding, genetic drift) of habitat fragmentation should be carefully evaluated in the Sardinian wild boar, in order to promote a sustainable management of its endemic genetic diversity.

## CONFLICT OF INTEREST

The authors have no conflicts of interest to declare.

## AUTHOR CONTRIBUTIONS

**Roberta Lecis:** Conceptualization (supporting); Data curation (equal); Methodology (equal); Software (lead); Writing – original draft (lead). **Olivia Dondina:** Conceptualization (supporting); Methodology (equal); Software (lead); Writing – original draft (equal). **Valerio Orioli:** Conceptualization (supporting); Methodology (equal); Software (equal); Writing – original draft (equal). **Daniela Biosa:** Data curation (equal); Investigation (equal); Writing – original draft (equal). **Antonio Canu:** Data curation (equal); Investigation (equal); Methodology (equal); Writing – review & editing (equal). **Giulia Fabbri:** Methodology (supporting); Software (equal); Writing – review & editing (equal). **Laura Iacolina:** Data curation (equal); Investigation (equal); Writing – review & editing (equal). **Antonio Cossu:** Data curation (supporting); Investigation (supporting); Writing – review & editing (equal). **Luciano Bani:** Conceptualization (supporting); Methodology (supporting); Supervision (equal); Writing – review & editing (equal). **Marco Apollonio:** Conceptualization (supporting); Funding acquisition (lead); Supervision (equal); Writing – review & editing (equal). **Massimo Scandura:** Conceptualization (lead); Data curation (equal); Funding acquisition (lead); Methodology (lead); Project administration (lead); Writing – original draft (equal); Writing – review & editing (equal).

## Supporting information

Appendix S1‐S2

## Data Availability

Microsatellite genotype data are deposited in the OSF public repository (https://doi.org/10.17605/OSF.IO/Y3HFS).
