# Supplementary material for: Main roads and land cover shaped the genetic structure of a Mediterranean island wild boar population
Source: Ecol Evol. 2022 Apr 6;12(4):e8804. doi: 10.1002/ece3.8804 (PMC8986547; doi:10.1002/ece3.8804)
Supplement: Supplementary file 1 — Appendix S1‐S2 [file ECE3-12-e8804-s001.docx]

**APPENDIX 1**. Results showing the most likely number of subpopulations identified by STRUCTURE in the original dataset including 568 samples (including reference continental wild boars and domestic pigs). a) Triangles connected by a solid line represent values of ΔK associated with different values of K, calculated across 10 runs in STRUCTURE according to Evanno et al. (2005) by the web-based application Structure Harvester, while dots represent L(K). b) Pie charts representing the individual membership of the 318 sampled wild boar to the four inferred clusters at K=4, representing Sardinian wild boars (cluster II and IV, light and dark blue), continental wild boars (cluster I, yellow) and domestic pigs (cluster III, black).

a) b)

**APPENDIX 2**. Results showing the most likely number of subpopulations identified by STRUCTURE in the purged dataset of 270 ‘pure’ Sardinian wild boar. a) Triangles connected by a solid line represent values of ΔK associated with different values of K, calculated across 10 runs in STRUCTURE according to Evanno et al. (2005) by the web-based application Structure Harvester, while dots represent L(K). b) Pie charts representing the individual membership of the 270 wild boar to the five inferred clusters at K=5, representing respectively the subpopulations NW (orange), CW (blue), SW (green), SE (purple), and NCE (yellow).

a) b)
